# McKillip Veterinary College

**Published:** 1896-07

**Authors:** 


McKillip Veterinary College, in its announcement for the coming collegiate year, will have added to her corps of teachers as Professor of Physiology, E. M. Reading, A.M., M.D., succeeding J. H. Honan, D.V.S., M.D., resigned. E. M. Broeckin, V.M.D., will fill the Chair of Histology and Microtechnology. Elmer E. Critchfield, western editor of the *National Stockman and Farmer*, has been added to the staff of teachers on Breeding and Hygiene. Its introductory pages strongly testify its unqualified belief in a three-years' course. The lecture fees have been reduced from one hundred to seventy-five dollars each year. An outline of the entire course, examinations, buildings, etc., is given in the catalogue, with illustrations of the various rooms, laboratories, and hospital wards.

